# Soluble Urokinase Receptors in Focal Segmental Glomerulosclerosis: A Review on the Scientific Point of View

**DOI:** 10.1155/2016/2068691

**Published:** 2016-07-18

**Authors:** Andreas Kronbichler, Moin A. Saleem, Björn Meijers, Jae Il Shin

**Affiliations:** ^1^Medical University Innsbruck, Anichstraße 35, 6020 Innsbruck, Austria; ^2^Children's and Academic Renal Unit, University of Bristol, Dorothy Hodgkin Building, Bristol BS2 8BJ, UK; ^3^Department of Microbiology and Immunology, KU Leuven and Department of Nephrology, UZ Leuven, 3000 Leuven, Belgium; ^4^Department of Pediatric Nephrology, Yonsei University College of Medicine, Severance Children's Hospital, Seoul 120-752, Republic of Korea

## Abstract

Focal segmental glomerulosclerosis (FSGS) is one of the primary glomerular disorders in both children and adults which can progress to end-stage renal failure. Although there are genetic and secondary causes, circulating factors have also been regarded as an important factor in the pathogenesis of FSGS, because about 40% of the patients with FSGS have recurrence after renal transplantation. Soluble urokinase-type plasminogen activator receptor (suPAR) is a soluble form of uPAR, which is a membrane-bound protein linked to GPI in various immunologically active cells, including podocytes. It has recently been suggested as a potential circulating factor in FSGS by in vitro podocyte experiments, in vivo mice models, and human studies. However, there have also been controversies on this issue, because subsequent studies showed conflicting results. suPAR levels were also increased in patients with other glomerular diseases and were inversely correlated with estimated glomerular filtration rate. Nevertheless, there has been no balanced review on this issue. In this review, we compare the conflicting data on the involvement of suPAR in the pathogenesis of FSGS and shed light on interpretation by taking into account many points and the potential variables and confounders influencing serum suPAR levels.

## 1. Background

Focal segmental glomerulosclerosis (FSGS) is a primary glomerular disorder with 50% of patients progressing to end-stage renal disease (ESRD) in those unresponsive to treatment [1–3]. FSGS can be divided into primary and secondary forms, but overlap of clinical and histologic features hampers differentiation in some cases [2]. It is considered to be a lesion with diverse clinical features and different pathophysiologic mechanisms and response to treatment [1–3].

Recent evidence shows that FSGS is mainly a “podocytopathy” with several podocyte-related molecules implicated in development and course of the disease, which is supported by insights into genetics from hereditary forms [4–6]. Circulating factors may be directly implicated in the pathogenesis of FSGS, since about 40% of the patients with primary FSGS have recurrence after kidney transplantation (KT), which may be higher in children than in adults, and significant progress of their pivotal role in the pathogenesis of primary FSGS has been achieved recently [7, 8].

Soluble urokinase-type plasminogen activator receptor (suPAR) has recently been suggested as a potential circulating factor in FSGS [9–13]. However, there have also been controversies on this issue, because suPAR levels were also increased in those with other glomerular diseases and were inversely correlated with estimated glomerular filtration rate (GFR) [14–24].

To resolve these discrepancies, we discuss current knowledge regarding the role of suPAR in the pathogenesis of primary FSGS and compare the conflicting data on this issue by taking into account the potential variables influencing serum suPAR levels with a balanced review.

## 2. The Role of Circulating Permeability Factors in FSGS

A role of a circulating factor in the etiopathogenesis of FSGS has first been proposed in 1972, when Hoyer and colleagues described a case series of patients with recurrent FSGS after KT [25]. Risk factors for disease recurrence include younger age, heavy proteinuria, higher baseline creatinine at the onset of the disease, and rapid progression to ESRD [26, 27]. Biopsies obtained from patients with recurrent FSGS resemble the same histologic subtype in a majority of patients. Plasmapheresis can remove the circulating factor and achieve remission in a subset of children and adults with FSGS [26, 28].

The putative circulating factor of patients with recurrent FSGS appeared to be bound to protein A and hydrophobic-interaction columns [29] and further investigations suggested the molecular mass of this factor to be around 30–50 kDa. Injection of supernatant from FSGS sera revealed threefold increased proteinuria in rats after 6 to 24 hours [30].

Onset of proteinuria after exposure to the circulatory factor could be influenced by several components, that is, apolipoproteins which might prevent glomerular albumin permeability after incubation with FSGS sera [31]. Undefined components of normal sera could prevent the increase of glomerular albumin permeability in cultured rat glomeruli [32]. Likewise, application of galactose might diminish glomerular albumin permeability in recurrent FSGS, indicating high affinity of the circulating factor for galactose [33, 34].

Transmission of the glomerular permeability factor from a mother to her unborn child further highlights the pathogenic role of a circulating permeability factor [35]. There have been several factors which have been proposed as potential candidates in the pathogenesis of primary FSGS such as vasodilator stimulated phosphoprotein (VASP) [36] or cardiotrophin-like-cytokine-1 (CLC-1) [7, 37]. Although not proved in FSGS, protein tyrosine phosphatase receptor-O (PTPRO) was suggested to increase glomerular albumin permeability [7, 37]. suPAR has recently been suggested as a potential circulating factor in FSGS by Wei et al. [9].

## 3. uPAR and suPAR

**Figure 1:**
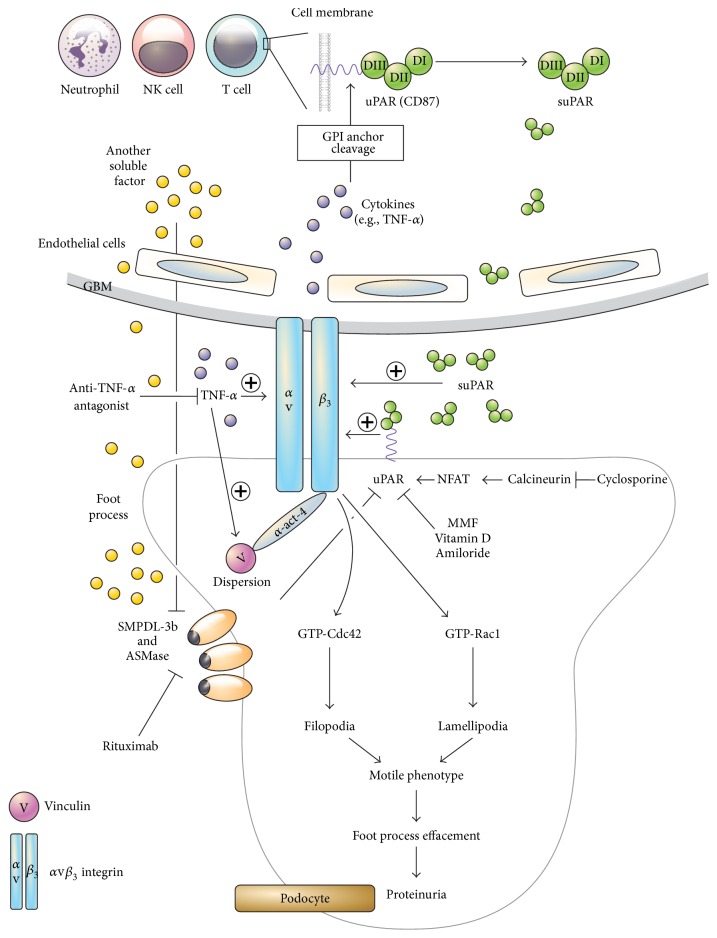
A hypothesis for the pathogenesis of suPAR-mediated FSGS. suPAR is formed from various immune cells after cleavage of GPI anchor by cytokines such as TNF-*α*. suPAR can activate *α*v*β*_3_ integrin of podocytes. TNF-*α* can directly activate podocyte *α*v*β*_3_ integrin and vinculin. Another serum factors might decrease SMPDL3b in podocytes, causing *α*v*β*_3_ integrin activation through increased binding of suPAR/uPAR and *α*v*β*_3_ integrin. Cdc42 and Rac1 can be activated by uPAR-*α*v*β*_3_ integrin signaling and the podocyte actin cytoskeleton shifts from a stationary to a motile phenotype, thereby causing foot process effacement and proteinuria. uPAR-*α*v*β*_3_ integrin signaling in podocytes can be blocked through various pathways. NK, natural killer; uPAR, urokinase-type plasminogen activator receptor; suPAR, soluble urokinase-type plasminogen activator receptor; GPI, glycosylphosphatidylinositol; TNF, tumor necrosis factor; NFAT, nuclear factor of activated T cells; MMF, mycophenolate mofetil; GTP, guanosine-5′-triphosphate; SMPDL, sphingomyelin phosphodiesterase acid-like; ASMase, acid sphingomyelinase.

The urokinase-type plasminogen activator (uPA) system is composed of a protease, a receptor (uPAR), and inhibitors [38]. uPAR was cloned in 1990 [39] and is a membrane-bound 45–55 kDa protein with three domains (D_I_, D_II_, and D_III_) linked to glycosylphosphatidylinositol (GPI) [38]. It is present in various immunologically active cells, such as neutrophils, lymphocytes, monocytes, macrophages, activated T cells, endothelial cells, megakaryocytes, tumor cells, and podocytes (Figure 1) [9, 38, 40–43]. uPAR can bind to various ligands such as uPA, vitronectin, and integrins [44]. Upon binding of uPA to its receptor (uPAR) it mediates various cellular activities such as adhesion, migration, differentiation, and proliferation [38]. In podocytes, uPAR is one of the pathways capable of activating *α*v*β*_3_ integrin promoting cell motility and activation of small GTPases, such as Cdc42 and Rac1, which can lead to podocyte contraction, shifting from a stationary to motile phenotype and leading to foot process effacement and proteinuria (Figure 1) [45].

If uPAR is cleaved from the cell surface, a soluble form of uPAR (suPAR) is released [38, 46]. Full-length suPAR (suPAR_I–III_) can be cleaved into another two soluble forms with different biologic properties, suPAR_II-III_ and suPAR_I_ [38]. The I–III portion of suPAR can compete with uPAR_I–III_ for uPA binding [47]. suPAR can be found in various body fluids including blood, plasma, serum, urine, saliva, and cerebrospinal fluid (CSF) in different concentrations [22, 48, 49] and have similar functions as uPAR [38].

suPAR can be measured with a monoclonal antibody double sandwich enzyme-linked immunosorbent assay (ELISA) method using commercially available kits (e.g., R&D Systems, Minneapolis, MN, USA; suPARnostic*™*, Virogates, Copenhagen, Denmark), but only R&D Systems were used in all the suPAR studies in FSGS. In healthy individuals, suPAR levels are stable in blood and urine and the whole molecule of suPAR was documented in serum and two truncated soluble forms of the entire molecule (suPAR_I_ and suPAR_II-III_) in the urine [50]. Elevated plasma or serum suPAR levels have been demonstrated in patients with various diseases, such as cancer, sepsis, systemic inflammatory response syndrome, and cardiovascular disease [51–55] and have been shown to be associated with a poor clinical outcome. The fact that suPAR is elevated by a large number of diseases, in particular liver and kidney diseases, makes it unlikely that suPAR can ever be used as a diagnostic biomarker (for FSGS or other diseases).

In summary, uPAR is a membrane-bound protein linked to GPI in various immunologically active cells, including podocytes, and is released as suPAR.

## 4. The Role of uPAR Signaling and Integrin Activation in Podocytes and Proteinuric Kidney Diseases

Podocyte foot processes contain an actin cytoskeleton, which is connected to the glomerular basement membrane by *α*3*β*_1_ and *α*v*β*_3_ integrin as well as *α* and *β*-dystroglycans [56]. Induction of uPAR signaling in podocytes leads to foot process effacement and urinary protein loss by lipid-dependent activation of *α*v*β*_3_ integrin [45]. Conversely, blocking of *α*v*β*_3_ integrin also reduced podocyte motility in vitro and lowered proteinuria in mice [45]. Also, mice lacking uPAR (plasminogen activator, urokinase receptor,* PLAUR* ^−/−^) were protected from lipopolysaccharide- (LPS-) mediated proteinuria but developed disease after expression of a constitutively active *α*v*β*_3_ integrin, suggesting the pathogenic role of uPAR signaling in the pathogenesis of proteinuric kidney diseases including FSGS [9, 45].

Recently, other groups provide further supportive evidence [57–62]. Inducible podocyte-specific expression of constitutively active nuclear factor of activated T cells (NFATc1) increased podocyte uPAR expression by binding to the* PLAUR* gene promoter [57]. Pathological uPAR signals in podocytes were independent of T cells and affected cell motility via activation of *β*_3_ integrin. This could be blocked by cyclosporine, NFAT-siRNA, or NFAT inhibitor in animal models of glomerular diseases such as LPS-treated or 5/6 nephrectomized rats [57]. Rapamycin could promote podocyte migration through the upregulation of uPAR, providing a new mechanism of rapamycin-associated proteinuria [58]. Conversely, several drugs targeting podocyte uPAR expression, such as vitamin D, amiloride, or mycophenolate mofetil (MMF), have recently been shown to be effective in reducing proteinuria in various experimental models, such as FSGS or lupus mice [59–61]. Recently, it was reported that rituximab might also inhibit uPAR-*β*_3_ integrin signaling via modulation of sphingomyelin phosphodiesterase acid-like 3b (SMPDL-3b) [62]. These cellular events are well summarized in Figure 1. In addition to uPAR-integrin signaling, podocyte vascular endothelial growth factor (VEGF)-A regulates *α*v*β*_3_ integrin signaling in the glomerulus, and podocyte VEGF knockdown disrupts *α*v*β*_3_ integrin activity [63].

In summary, uPAR-*α*v*β*_3_ integrin signaling is an important pathway in the pathogenesis of proteinuria in various kidney diseases.

## 5. The Role of suPAR in Podocytes and FSGS

Wei et al. reported that circulating suPAR could activate *β*_3_ integrin in a similar manner to membrane-bound uPAR in podocytes by using the methods of coimmunoprecipitation of suPAR and *β*_3_ integrin and the activating epitope-recognizing antibodies such as the *β*_3_ integrin-specific antibody AP5 [9]. They found that the incubation of podocytes with recombinant suPAR strongly induces AP5 signal in a specific pattern highlighting areas of focal adhesions, which are known to be the location of *β*_3_ integrin. This effect was blocked by a blocking specific uPAR antibody [9].

They established three different mouse models [9]. The first was* PLAUR* ^−/−^ mice: high-dose recombinant mouse suPAR_I–III_ induced proteinuria and foot process effacement, prominent deposits of suPAR on podocytes as well as increased podocyte *β*_3_ integrin activity [9]. The second model was hybrid-transplant mice modeling endogenous suPAR release in which a kidney from* PLAUR* ^−/−^ mice was transplanted in a wild-type mouse and proteinuria developed after LPS-induced suPAR production, indicating that circulating suPAR may be able to activate *β*_3_ integrin independently of uPAR [9]. The third model was genetically engineered wild-type mice. Wild-type mice were injected with a suPAR_I-II_-producing plasmid into their skin, which caused increased serum suPAR concentrations and FSGS-like lesions with proteinuria. A plasmid with a point mutation in the D_II_ domain induced synthesis of suPAR in wild-type mice. However, this was unable to bind to *β*_3_ integrin and did not induce proteinuria [9].

In their study, serum suPAR concentrations were significantly elevated in patients with FSGS compared to those with minimal change disease (MCD, either in relapse or remission), membranous nephropathy (MN), preeclampsia, and healthy subjects [9]. suPAR concentrations correlated with the activity of podocyte *β*_3_ integrin and inhibition of suPAR by cycloRGDfV or antibodies specific against uPAR and plasmapheresis could lower AP5 activity on podocytes [9]. They also found a predominant suPAR fragment at ~22 kDa and the other two forms at ~45 and 40 kDa, albeit at much lower expression levels [9]. However, serum uPA concentrations did not differ between FSGS and other glomerular diseases [9].

In summary, Wei et al. [9] reported an important role of suPAR in the pathogenesis of FSGS with in vitro podocytes, 3 mice models of FSGS, and FSGS patients.

## 6. Subsequent Clinical Observations Supporting the Pathogenic Role of suPAR in FSGS

In two large cohorts, circulating suPAR levels were elevated in 84.3% (North American) and 55.3% (the European PodoNet) of the FSGS patients compared with 6% of controls [10]. Inflammation did not account for this difference [10]. Serum suPAR levels were also increased in FSGS compared to other glomerulopathies such as MCD or MN and healthy subjects [9–13]. However, serum suPAR levels did not differ between primary and secondary FSGS [12]. One study showed that elevated suPAR levels in primary FSGS were not merely attributable to decreased estimated eGFR, because suPAR levels of primary FSGS were still significantly higher than MCD or MN after adjusting for renal function [12]. One study reported the usefulness of suPAR measurements in predicting steroid response in patients with primary FSGS [13].

In 2001, Kemper et al. reported an infant with transient proteinuria born to a mother with FSGS [35] and a recent reanalysis of stored samples revealed highly elevated suPAR levels in both the mother and the newborn [64]. Extracorporeal treatment reduced suPAR, podocyte *β*_3_ integrin activation, podocyte effacement, and proteinuria in recurrent FSGS patients in accordance with reduction of suPAR levels [65, 66].

Recently, it was reported that urinary suPAR levels of patients with primary FSGS were significantly higher compared to those with MCD, MN, and secondary FSGS and normal subjects [67] and positively correlated with 24-hour urinary protein excretion in primary FSGS. During follow-up, urinary suPAR levels decreased in patients with complete remission. After incubation of human podocytes with urine obtained from patients with primary FSGS, the AP5 signal was induced and it could be reduced by a blocking antibody to uPAR [67].

## 7. Clinical Observations Not Supporting the Pathogenic Role of suPAR in FSGS

Although increased suPAR levels resulted in FSGS-like glomerular lesions and proteinuria in* PLAUR* ^−/−^ mice [9], the pathogenic effects of suPAR were not observed in wild-type mice, in which proteinuria or podocyte foot process effacement did not occur despite glomerular suPAR deposition [23, 68]. suPAR concentrations did not distinguish patients with FSGS from other glomerular histopathologies such as MCD, MN, IgA nephropathy, and lupus nephritis or nonglomerular chronic kidney disease (CKD), suggesting that suPAR might be involved in the pathogenesis of various renal diseases as a nonspecific marker or impaired glomerular integrity might affect its clearance [14–24]. Moreover, eGFR was the strongest determinant of suPAR concentration [14–24]. Therefore, more work is warranted and justified to explore the role of suPAR in FSGS and other glomerular diseases.

Wei et al. reported that pretransplant serum suPAR levels predicted posttransplant recurrence of FSGS [9], whereas several other investigators found no evidence that serum suPAR was a specific marker for FSGS recurrence [18, 22, 69]. Although there have been studies demonstrating a decrease of suPAR after the induction of remission [10, 12, 65, 66], others demonstrated similar serum levels regardless of therapeutic response [15, 18, 70]. Recently, it was demonstrated that urinary suPAR levels rather than serum suPAR levels might be helpful in the early identification of patients at high risk of posttransplant FSGS recurrence [22].

From an epidemiologic point of view, statistical validation is also one of the important steps to be a reliable surrogate biomarker from biomarker discovery to clinical utility [71]. A biomarker for clinical use needs good sensitivity and specificity (e.g., ≥0.9) and good positive and negative predictive value. However, previous studies on suPAR had many problems in the study designs, sample collection, and statistical analysis techniques. Most studies on suPAR were conducted in a retrospective design and the selection of healthy controls was not matched for age, sex, and other parameters influencing suPAR levels. With regard to statistics, most studies did not perform multiple logistic regression analysis to find an independent predictor and receiver operating characteristics (ROC) curve analysis to calculate sensitivity and specificity, an essential prerequisite to be a biomarker, but simply presented the differences of suPAR levels among groups. Furthermore, some studies did not present the mean ± standard deviation (SD), hampering the meta-analysis of several suPAR studies. If there are many factors influencing suPAR levels, an individual patient data meta-analysis and propensity score matching would be important statistical methods to elucidate whether suPAR could be a reliable surrogate biomarker in this field.

There has also been interest in reproducibility concerns in medicine [72]. In addition to registration of the study design (i.e., http://www.clinicaltrials.gov/), the adoption and following of the guidelines such as STrengthening the Reporting of OBservational studies in Epidemiology (STROBE) for cohort and case control studies would increase the reproducibility and reliability in human studies [73]. Irreproducibility of preclinical animal data can also lead to the failure of human clinical trials and ARRIVE (Animal Research: Reporting of In Vivo Experiments) for animal studies which may improve the quality of animal research and increase transparency and reproducibility [74].

## 8. Areas of Uncertainty concerning Preclinical Studies Investigating suPAR in FSGS

### 8.1. Different Mice Models Used in suPAR Studies

The search for a good model of human recurrent FSGS is indeed a key to future studies and has yet to be developed. In our opinion, the most appropriate mouse model to study FSGS-like lesions should be determined. The animal models described are specific to studies of suPAR and may not be useful if other factors or cofactors are responsible for this disease. While Wei et al. used* PLAUR* ^−/−^ mice and genetically engineered wild-type mice with injection of a suPAR_I-II_-producing plasmid, most others performed studies with wild-type mice [9]. Maas et al. pointed out that wild-type nonproteinuric mice used in the original study already exhibited suPAR levels of approximately 3,000 pg/mL at baseline before activation of the plasmid [75]. After initiation of recombinant suPAR expression, mice developed proteinuria along with a sharp rise in urinary suPAR excretion and rise of serum suPAR. However, these mice with high baseline suPAR might be prone to develop FSGS [9]. In contrast, Cathelin et al. used wild-type C57BL/6J and 129S2/SvPas mice and intravenous administration of suPAR could not cause proteinuria despite massive suPAR deposits in the glomeruli in these mice and did not aggravate proteinuria in LPS-treated C57BL/6J mice [68]. They addressed that if the genetic ablation of* PLAUR* in the kidney is the cause of the effect observed on proteinuria, it is difficult to translate this finding to the pathogenesis of FSGS in humans. They also pointed out that the plasmid used in genetically engineered WT mice by Wei et al. [9] encodes a truncated mRNA splice variant of mouse uPAR covering the first 133 residues of the full-length receptor and this particular splice mRNA transcript may not be translated into a folded protein product in vivo because it encodes only one and one half LU-domain [68].

Spinale et al. also used wild-type and a transgenic mouse model continuously inducing hepatic suPAR expression (D1D2D3) in which proteinuria did not develop despite an increase of suPAR [23]. They pointed out that Wei et al. [9] injected a commercially available mouse recombinant Fc-fusion protein in short-term experiments that coupled a human IgG1 Fc-domain to mouse uPAR lacking a GPI-linkage motif and it has not been determined whether IgG1 Fc domain containing protein engendered complement fixation-dependent glomerular injury [23]. They also addressed that Wei et al. [9] used a mouse suPAR cDNA fragment obtained from a purchased cDNA clone (IMAGE cDNA clone 3158012) containing a retained intron 4 (uPAR-intron 4) and the mouse splice variant encoding uPAR-intron 4 is rare and the expression of the protein associated with this variant has not been reported [23]. In addition, they indicated that Wei et al. [9] reported the creation of a control mouse suPAR construct with an E134A mutation intended to abrogate binding of mouse suPAR to *β*_3_ integrin, but a similar *β*_3_ integrin binding motif has not been described in mouse uPAR [23].

In various inflammatory conditions, an increase in serum suPAR levels has been reported which was not associated with proteinuria [38, 48, 53, 54, 76], suggesting that suPAR itself may not be sufficient to induce nephrotic proteinuria as in wild-type mice.

In our opinion, however, the threshold of serum suPAR at which proteinuria and a FSGS-like lesion develop should be determined both in wild-type mice [23, 68] and in* PLAUR* ^−/−^ mice [9], requiring further dose-response experiments in different animal models of FSGS in the future. We speculate that much higher doses and more prolonged administration of suPAR could be used to determine the threshold in wild-type mice. In addition, with regard to the measurements of suPAR levels in mice, Cathelin et al. did not measure serum suPAR levels in suPAR-treated mice [68]. Wei et al. described that murine suPAR was evaluated by an in-house ELISA kit [9] and Spinale et al. reported that suPAR concentration in mouse serum was measured with a kit from R&D Systems (Minneapolis, MN) (DY531) which has been validated by the manufacturer for detection of mouse suPAR in cell supernatant [23]. The measurements of suPAR levels by ELISA methods in mice should be unified and further validated in both wild-type and genetically engineered mice in future studies for the comparison.

In summary, we cannot say that suPAR is not involved in the pathogenesis of FSGS in mice, because different experimental models were used.

### 8.2. In Vitro suPAR Studies on Podocytes

Wei et al. demonstrated that recombinant suPAR activates *β*_3_ integrin (AP5 staining) in a similar manner to membrane-bound uPAR in podocytes, which was blocked by an antibody specific to uPAR. Currently, however, no further study has repeated and validated these findings [9]. Therefore, factors mediating suPAR-induced activation of *α*v*β*_3_ integrin need to be elucidated. Jefferson and Alpers speculated that activation of *α*v*β*_3_ signaling by suPAR might require additional modifying factors such as loss of podocyte protective factors or an underlying permissive genetic background [77].

Recently, Yoo et al. [78] reported that SMPDL-3b is an important regulator of suPAR-induced activation of *α*v*β*_3_ integrin signaling in podocytes. SMPDL-3b interferes with binding of suPAR/uPAR and *α*v*β*_3_ integrin, attenuating *α*v*β*_3_ integrin activation. They showed that SMPDL-3b expression is decreased in glomeruli of patients with recurrent FSGS and FSGS sera-treated podocytes exhibited decreased SMPDL-3b expression [78]. Therefore, high suPAR levels could increase podocytic *α*v*β*_3_ integrin activation in FSGS patients with nephrotic syndrome, while intact SMPDL-3b expression in podocytes of normal subjects might offset *α*v*β*_3_ integrin activation by inflammation-driven high suPAR. Notably, they demonstrated that high suPAR in the presence of increased podocyte SMPDL-3b levels in diabetic nephropathy did not activate *α*v*β*_3_ integrin but led to RhoA activation and podocyte apoptosis, indicating different effector pathways in suPAR signaling [78].

We should also note that FSGS sera-treated podocytes exhibited decreased SMPDL-3b expression but suPAR itself did not modulate SMPDL-3b expression levels [78]. Therefore, in our opinion, other unknown factors in FSGS might reduce the SMPDL-3b expression in injured podocytes, increasing the effect of suPAR on podocytes, and this has to be clarified (Figure 1).

In summary, the role of suPAR in activating *β*_3_ integrin in podocytes has not been repeated in other groups and further validation is necessary.

## 9. Areas of Uncertainty concerning the Sources of suPAR

As mentioned above, various suPAR fragments exist with different characteristics and whether or not the “true” circulatory factor is a cleaved suPAR isoform remains obscure. Wei et al. found a predominant suPAR fragment at ~22 kDa [9]. The importance of different suPAR domains was highlighted by the finding that suPAR administration in mice producing suPAR D_I_ and D_II_ domains induced albuminuria [9]. Trachtman et al. considered that it is likely that all forms of suPAR can bind to *α*v*β*_3_ integrin, but subsequent activation might vary depending on the specific form of suPAR [79]. Maas et al. speculated that vitronectin-binding capacity of suPAR fragments might determine the activity as a FSGS factor [75]. In addition, it should be considered that a glycosylation status of suPAR may be causative of inducing proteinuria in primary FSGS [80]. Because the currently available ELISA kits can detect full-length glycosylated suPAR only [80], characterization of the different isoforms and their biologic activity is clearly warranted and should be addressed by further studies, using novel kits. In addition, the levels of different isoforms in active FSGS should be clearly clarified, as the original clinical studies are difficult to reconcile if it is only a specific isoform that is biologically active.

In summary, isoforms and glycosylation status of suPAR should be considered and detection kits should be developed in the future.

## 10. Areas of Uncertainty concerning suPAR Serum Determinants

The time of specimen collection may influence suPAR levels [81]. However, no differences between serum and plasma suPAR levels were observed when samples were kept at room temperature for a few hours, but suPAR levels increased after an observation time of 72 hours. Repeated freeze and thaw cycles had no influence on suPAR levels [82]. Therefore, uniform specimen collection and only short-term storage at room temperature should be targeted.

The relevance of demographics in the interpretation of serum suPAR has to be highlighted. In healthy adults, mean suPAR concentrations of approximately 2,000–4,000 pg/mL have been reported [52]. Although there have only been few studies on potential factors influencing suPAR levels in the normal population, higher suPAR levels were found in women, smokers, older subjects, and Africans (compared to Caucasians) [52, 83]. Therefore, there is a strong need to include these confounding factors in future studies investigating the role of suPAR in FSGS.

Markers of systemic inflammation (i.e., C-reactive protein (CRP) or erythrocyte sedimentation rate (ESR)) should be determined as well, since inflammation per se can affect suPAR levels [16, 18, 52, 76]. A positive correlation between suPAR and either CRP or ESR levels was shown in patients with rheumatoid arthritis [76]. No correlation has been reported between CRP and suPAR levels in FSGS so far [10, 13, 17]. However, there were correlations between suPAR and high sensitivity CRP (hsCRP) levels or log CRP [16, 18]. There remains uncertainty towards the value of CRP as inflammatory marker in nephrotic disease, but the use of hsCRP may be a more appropriate measure to correlate with suPAR [18] and might be used in further studies.

In most studies, eGFR is one of the strongest determinants of suPAR concentration [14–24], but these findings could not be confirmed in two studies [9, 13]. Accumulation of suPAR in patients at low GFR may obfuscate FSGS-induced suPAR accumulation [84]. Information about clearance, production, and release of suPAR in patients with preserved or impaired kidney function is lacking. Although several factors such as inflammatory cytokines are increased in chronic kidney disease (CKD) [85–87], we still do not know whether these changes may lead to increased suPAR levels or not. In one study, hemodialysis patients had very high suPAR levels. The median suPAR in these patients was 12.3 ng/mL (controls 3.2 ng/mL, suPARnostic assay), which makes this group of patients quite unique with regard to the extremely high suPAR level [88].

In summary, suPAR serum determinants (collection time of blood, ethnic differences, systemic inflammation, eGFR, and metabolism of suPAR) should be considered in interpreting the results.

## 11. Areas of Uncertainty concerning suPAR in FSGS

Most studies did not correlate suPAR levels with the different histopathological variants of primary FSGS. Others reported no differences among FSGS subtypes [11, 13, 89]. However, Huang et al. found that suPAR levels were higher in tip-lesion FSGS, followed by not otherwise specified and cellular variant [12], whereas urinary suPAR levels were highest in patients with the cellular variant [67]. They found no differences in suPAR levels between primary and secondary FSGS [12], but Segarra et al. found an association of suPAR levels with the diagnosis of primary FSGS after adjusting for age, renal function, and presence of nephrotic syndrome [89]. We propose that multiple cohorts should be reanalyzed according to the Columbia classification of primary FSGS to highlight potential differences between the variants and this should be considered in future studies as well.

Circulating factors such as suPAR have their most significant role in patients at risk of recurrent FSGS after KT. However, most studies did not measure suPAR levels in recurrent versus nonrecurrent FSGS [11–24]. Another limitation of research may be misclassification of FSGS cases, since distinction between primary and secondary FSGS may not be feasible in all patients. Wei et al. reported that FSGS patients with an NPHS2 mutation had higher suPAR levels than those without [10], but Maas et al. questioned this result, because it is in contrast with the observation that recurrences of FSGS occur most frequently in patients with nongenetic, primary FSGS [75]. Jungraithmayr et al. reported that none of the 11 patients with homozygous or compound heterozygous NPHS2 mutations developed recurrent FSGS after KT compared with 45% of patients without mutations [90]. Therefore, higher suPAR levels in FSGS patients with an NPHS2 mutation might not influence the risk of recurrence after KT. The incidence of NPHS2 mutations seems to be very rare in Asian countries [91] and therefore suPAR studies from Asian countries might rarely include FSGS patients with podocin mutations. Early studies suggested that a few patients with NPHS2 “mutations” do develop FSGS recurrence after KT [92, 93], but subsequent information suggests that these may not be true disease causing variants.

In summary, the role of suPAR should be interpreted in the context with recurrent FSGS after KT, which is considered to be circulating factor-mediated.

## 12. Areas of Uncertainty concerning Immunosuppression and suPAR Levels

The use of immunosuppressive drugs (the kinds, the cumulative dose, and the duration of immunosuppressants) at the time of suPAR measurements should be considered. Although most studies did not describe this crucial parameter, suPAR in the European PodoNet cohort comprising steroid-resistant children and adolescents revealed lower levels in the MMF-treated group, while no difference was observed in patients with or without calcineurin inhibitor treatment [10]. Wada et al. showed that suPAR levels were significantly lower in FSGS patients with normal renal function who were treated with steroids or immunosuppressants than in those without, but the use of steroids/immunosuppressants was not predictive of suPAR levels in those with glomerular diseases [17]. Sinha et al. reported that serum suPAR levels did not correlate with the duration of immunosuppressive therapy [18]. A recent study showed that serum suPAR levels decreased after MMF therapy, while they increased after cyclosporine treatment in children with MCD and frequently relapsing steroid-sensitive nephrotic syndrome with normal renal function, suggesting that immunosuppressants per se might have diverse effects on suPAR levels [94].

The immunologic nature of suPAR has to be considered, because it may be influenced secondarily by immune activation. There might be an influence of cytokines such as TNF-*α* and interleukin-2 (IL-2) on suPAR [95–97]. Although not tested in FSGS, suPAR correlated with leukocyte count and TNF-*α* [95] and several suPAR isoforms with soluble TNF receptor-II in HIV-infected patients [96]. TNF-*α* was supposed to be critical for uPAR expression on platelets [98]. Park et al. recently speculated that IL-2 might be an important cytokine for the formation of suPAR from T cells and natural killer cells in FSGS [97].

Recently, Delville et al. [99] screened about 9000 antigens in pretransplant sera of FSGS and pretransplant elevation of anti-CD40 antibody (Ab) alone had the best correlation (78% accuracy) with recurrent FSGS risk after KT among seven Abs (CD40, PTPRO, CGB5, FAS, P2RY11, SNRPB2, and APOL2). Anti-CD40 Abs purified from the sera of recurrent FSGS patients were particularly pathogenic in human podocyte cultures and injection of anti-CD40/rFSGS Ab enhanced suPAR-mediated proteinuria in wild-type mice, but no sensitizing effect was noted in mice deficient in CD40 or in wild-type mice that received blocking Ab to CD40, supporting the fact that suPAR might be formed by immunologically mediated mechanisms in FSGS [99]. Therefore, further investigations are necessary to elucidate the role of various immunological molecules on podocytes, mice models of FSGS, and patients with FSGS.

More recently, recurrent FSGS after KT was successfully treated with immunosuppressive treatments with high-dose methylprednisolone and cyclosporine [100], potentially suppressing TNF-*α* and IL-2. A TNF-*α* driven pathway in primary FSGS has also been supported by findings of Bitzan et al. who reported a patient with recurrent and plasmapheresis-resistant FSGS with sustained partial remission of proteinuria after initiation of anti-TNF-*α* therapy despite discontinuation of plasmapheresis [101]. Moreover, they showed that activated podocyte *β*_3_ integrin by plasma from patients with FSGS recurrence could be reversed by blocking TNF-*α* in vitro (Figure 1) [101].

Therefore, in our opinion, increased circulating factors such as suPAR might reflect a secondary effect of immune activation in FSGS. Treatment suppressing cytokines such as TNF-*α* and IL-2 could induce remission possibly due to a decrease of the circulating factors. It was speculated that a cytokine such as TNF-*α* might have direct deleterious effects on podocytes in vivo [102] and this effect could be therapeutically targeted. However, the question remains whether TNF-*α* is the specific elusive “factor” or just one out of several factors affecting the glomerular filtration barrier or modulating the immune response [102]. We think that glomerular diseases like FSGS may develop when suPAR interacts with other factors that injure podocytes in a proposed multiple hit process. However, the precise effects of TNF-*α* or IL-2 on podocytes have not been elucidated leaving the opportunity that an inflammatory signal rather than suPAR is causing FSGS-type lesions. We encourage consideration of additive or synergic effects of several influences (circulatory factors, genetic susceptibility, and local immunologic changes) on the glomerular filtration barrier of which suPAR may be one such influence or may be pathologically altered by other cytokines.

In summary, suPAR may be an immunologically mediated molecule and immunosuppression can suppress the suPAR levels.

## 13. Conclusions and Future Perspectives

Circulating factors have been implicated in the pathogenesis of recurrent FSGS after KT [7, 8] and suPAR has been suggested as a potential candidate. Circulating suPAR could activate *β*_3_ integrin in a similar manner to membrane-bound uPAR in podocytes, but further validations are necessary to elucidate how much activation of *β*_3_ integrin is relevant to cause foot process effacement. suPAR could induce proteinuria in vulnerable mice models [9], but administration of suPAR to wild-type mice did not induce proteinuria [23, 68]. There might be a pivotal role of additional hits, such as increased TNF-*α* and IL-2 or decreased SMPDL-3b expression in podocytes in the development of proteinuria [78, 97, 101]. In the clinical setting, recurrent nephrotic range proteinuria in patients with nongenetic disease can be abrogated by intensive immunosuppression possibly due to repression of circulating factor formation, stabilization of the actin cytoskeleton (e.g., cyclosporine), or prevention of SMPDL-3b downregulation (e.g., rituximab) [62, 100, 103–106].

The conflicting results on suPAR levels between FSGS and other glomerular diseases or controls may be attributable to the heterogeneous nature of FSGS and various confounding factors as stated above. As stated above, clinical studies should be designed taking into account important covariates, for example, the GFR. Future studies should also be designed to formally evaluate the sensitivity, specificity, and predictive value of suPAR.

We summarized the different results on various suPAR studies in FSGS in Tables 1 and 2 and showed an integrative model for the pathogenesis of circulating factor-mediated FSGS in Figure 1. In our opinion, the data on the nature and biological effect of specific suPAR molecular fragments are incomplete so far. A single suPAR value is of no use as clinical biomarker and suPAR itself may not be sufficient to induce FSGS lesions. However, the downstream biological effects via *α*v*β*_3_ integrin may still be valid and suPAR might activate *α*v*β*_3_ integrin under other coexisting conditions such as reduced SMPDL-3b expression by other serum factors of FSGS or a costimulatory effect with TNF-*α*.

We recommend and encourage further investigations in this field to elucidate the role of various immunological molecules on podocytes, mice models of FSGS, and patients with FSGS. We suggest multicenter collaborative studies to clarify the controversies related to suPAR as circulatory factor in FSGS. We believe that understanding the immunology in this field may facilitate unraveling pathogenic mechanisms leading to FSGS.

## Figures and Tables

**Table 1:** Studies on suPAR in FSGS.

Pathogenic role of suPAR in FSGS	References	Nonpathogenic role of suPAR in FSGS	References
*In vitro study*			
suPAR activated *β*_3_ integrin in a similar manner to membrane-bound uPAR in podocytes	[9]	Not repeated by others	
Podocyte *β*_3_ integrin activation by suPAR was blocked by a blocking antibody specific to uPAR	[9]	Activated podocyte *β*_3_ integrin by plasma from patients with recurrent FSGS could be also reversed by blocking TNF-*α*	[101]

*In vivo mice study*			
High-dose recombinant mouse suPAR_I–III_ induced podocyte integrin *β*_3_ activation, proteinuria, and foot process effacement in a uPAR-knockout (*Plaur* ^−/−^) mice	[9]	Neither single-dose nor prolonged administration of recombinant suPAR induced albuminuria or podocyte foot process effacement despite massive suPAR deposits in the glomeruli in wild-type C57BL/6J and 129S2/SvPas mice	[68]
Proteinuria developed after LPS-induced suPAR production in a hybrid-transplant mice in which a kidney from uPAR-knockout (*Plaur* ^−/−^) mice was transplanted in a wild-type mouse	[9]	Coadministration of either monomeric or chimeric suPAR produced no additional effect; LPS-induced podocyte effacement and proteinuria in C57BL/6J mice	[68]
Injection of a suPAR_I-II_-producing plasmid in their skin led to increased serum suPAR concentrations and FSGS-like lesions with proteinuria in genetically engineered wild-type	[9]	Injection of Fc-chimeric suPAR to wild-type mice or continuous expression of suPAR from the liver in new transgenic mice did not induce proteinuria	[23]

*Human study*			
suPAR is increased in FSGS compared to other glomerulopathies and healthy subjects	[9–12]	suPAR is not increased in FSGS compared to other glomerulopathies	[14–21]
suPAR is more increased in recurrent FSGS after KT than in nonrecurrent FSGS	[9]	Not studied in other groups	
Pretransplant serum suPAR predicted recurrence of FSGS after KT	[9, 66]	Pretransplant serum suPAR did not predict recurrence of FSGS after KT	[18, 69]
		Rather, urine suPAR predicted recurrence of FSGS after KT	[22]
Increasing serum suPAR levels after KT predicted recurrence of FSGS	[9]	Serum suPAR levels did not increase at the time of FSGS recurrence after KT	[20]
Serum suPAR levels decreased after plasmapheresis or at remission of FSGS	[9, 10, 12, 65, 66]	Serum suPAR levels were similar regardless of FSGS recurrence after KT or between nephrotic state and remission of FSGS	[15, 18, 70]

uPAR, urokinase-type plasminogen activator receptor; suPAR, soluble urokinase-type plasminogen activator receptor; FSGS, focal segmental glomerulosclerosis; KT, kidney transplantation; TNF, tumor necrosis factor; LPS, lipopolysaccharide.

**Table 2:** Different results on various suPAR studies in FSGS and other glomerulopathies.

Negative results	References	Positive results	References
Serum suPAR levels did not correlate to eGFR	[9, 13]	Serum suPAR levels were inversely correlated with eGFR	[10, 14–24]
Serum suPAR levels did not correlate to CRP levels	[10, 13, 17]	Serum suPAR levels were positively correlated with CRP levels	[16, 18]
Serum suPAR levels are not influenced by immunosuppression	[17, 18]	Serum suPAR levels are influenced by immunosuppression	[10, 93]
Serum suPAR levels are not influenced by subtypes of FSGS	[11, 13, 89]	Serum suPAR levels are influenced by subtypes of FSGS	[12]
		Urine suPAR levels are influenced by subtypes of FSGS	[67]
Serum suPAR levels are not different between primary and secondary FSGS	[12]	Serum suPAR levels are higher in primary FSGS than in secondary FSGS	[89]
		Urine suPAR levels are higher in primary FSGS than in secondary FSGS	[67]
Serum suPAR levels did not predict response to steroids	[14, 18]	High serum suPAR levels predicted better response to steroids	[13]
High serum suPAR levels were not associated with acute tubulointerstitial lesions	[13]	High serum suPAR levels were associated with >50% interstitial fibrosis	[12]

suPAR, soluble urokinase-type plasminogen activator receptor; FSGS, focal segmental glomerulosclerosis; CRP, C-reactive protein; eGFR, estimated glomerular filtration rate.
